# Dynamics of *Schistosoma haematobium *egg output and associated infection parameters following treatment with praziquantel in school-aged children

**DOI:** 10.1186/1756-3305-5-298

**Published:** 2012-12-21

**Authors:** Katarina Stete, Stefanie J Krauth, Jean T Coulibaly, Stefanie Knopp, Jan Hattendorf, Ivan Müller, Laurent K Lohourignon, Winfried V Kern, Eliézer K N’Goran, Jürg Utzinger

**Affiliations:** 1 Department of Epidemiology and Public Health, Swiss Tropical and Public Health Institute, P.O. Box, CH–4002 Basel, Switzerland; 2 Center for Infectious Diseases and Travel Medicine, Department of Medicine, Albert-Ludwigs-University, Hugstetter Strasse 55, D-79106, Freiburg, Germany; 3 Centre Suisse de Recherches Scientifiques en Côte d'Ivoire, 01 BP 1303, Abidjan 01, Côte d'Ivoire; 4 University of Basel, P.O. Box, CH–4003 Basel, Switzerland; 5 Unité de Formation et de Recherche Biosciences, Université Félix Houphouët-Boigny, 22 BP 770, Abidjan 22, Côte d'Ivoire

**Keywords:** Schistosomiasis, *Schistosoma haematobium*, Praziquantel, Drug efficacy, Macrohaematuria, Microhaematuria, Proteinuria, Leukocyturia, School-aged children, Côte d’Ivoire

## Abstract

**Background:**

Praziquantel is the drug of choice in preventive chemotherapy targeting schistosomiasis. Increasing large-scale administration of praziquantel requires monitoring of drug efficacy to detect early signs of development of resistance. Standard protocols for drug efficacy monitoring are necessary. Here, we determined the optimal time point for praziquantel efficacy assessment against *Schistosoma haematobium* and studied the dynamics of infection parameters following treatment.

**Methods:**

Ninety school-aged children from south Côte d’Ivoire with a parasitologically confirmed *S. haematobium* infection were treated with a single oral dose of praziquantel (40 mg/kg) and followed up for 62 days post-treatment. Urine samples were collected on 23 schooldays during this period and were subjected to visual examination (macrohaematuria), urine filtration and microscopy (*S. haematobium* eggs) and reagent strip testing (microhaematuria, proteinuria and leukocyturia).

**Results:**

Observed cure and egg reduction rates were highly dependent on the time point post-treatment. Egg reduction rates were high (>97%) in weeks 3–9 post-treatment. Cure rates were highest in weeks 6 (92.9%) and 9 (95.0%) post-treatment. The prevalence of infection-associated parameters decreased after treatment, reaching a minimum of 2.4% in weeks 5 (proteinuria) and 7 (leukocyturia) post-treatment, and 16.3% at the end of week 8 (microhaematuria). Macrohaematuria disappeared between weeks 3 and 6 post-treatment.

**Conclusions:**

For monitoring praziquantel efficacy against *S. haematobium*, we recommend that the cure rate is assessed at week 6 post-treatment. The egg reduction rate can be evaluated earlier, from day 14 post-treatment onwards. Reagent strips are a useful additional tool for evaluating treatment outcomes in areas with high endemicity, preferably at weeks 5 and 6 post-treatment. The delayed decrease of microhaematuria confirms that lesions in the urinary tract persist longer than egg excretion post-treatment.

## Background

Schistosomiasis is caused by blood flukes of the genus *Schistosoma*. More than 200 million people are infected worldwide [1]. The largest proportion of infected people (>90%), live in sub-Saharan Africa, where schistosomiasis is considered a major public health problem [2-4]. Nevertheless, schistosomiasis is often given only token attention, similar to many other neglected tropical diseases [5-8]. Schistosomiasis is characterized by chronic inflammation around schistosome eggs that are trapped in host tissues. In the human host, *Schistosoma haematobium* is found in the veins of the small pelvis, causing urogenital schistosomiasis. Eggs of *S. haematobium* are usually excreted through the urinary tract. Typical signs of urogenital schistosomiasis are macro- and microhaematuria, proteinuria and leukocyturia [9,10]. School-aged children are at highest risk of infection and prevalence peaks between the ages of 8 and 15 years [9,11]. Chronic schistosomiasis is associated with anaemia, growth stunting and reduced physical fitness [12,13].

The most widely used diagnosis for *S. haematobium* is based on the microscopic detection of eggs in urine, using the filtration method [14,15]. Visual examination of urine samples for macrohaematuria, urine-albumin and haemoglobin level assessment with a photometer, and reagent strip testing for microhaematuria, proteinuria and leukocyturia can be used as indirect screening methods for urogenital schistosomiasis in areas with high endemicity [1,9,10,15-18].

The drug of choice for the treatment of schistosomiasis is praziquantel [9,19-21]. Praziquantel acts against all schistosome species and has a good safety profile [19-21]. The major shortcoming of praziquantel is its lack of efficacy against schistosomula, the young developing stages of the parasite [22,23]. There are currently no alternative treatment options to praziquantel, even though some substances have shown promising antischistosomal activity [15,20,24,25]. The global strategy for schistosomiasis control is preventive chemotherapy, where school-aged children (and other high-risk communities) are treated with a single oral dose of praziquantel (40 mg/kg) without prior individual diagnosis [6,15,26,27]. This strategy is going to scale; in 2010, for example, 33.5 million people were treated with praziquantel [28]. The large-scale administration of praziquantel without any backup drugs is of a considerable concern, should resistance to praziquantel emerge [21]. Hence, the efficacy of praziquantel against schistosomiasis needs careful monitoring [24].

To be able to promptly detect the emergence of resistance in schistosomes, standard protocols for drug efficacy monitoring are needed [29]. This includes the optimal time point post-treatment for measuring drug efficacy of praziquantel in urogenital schistosomiasis. Hence, we designed a study and assessed the dynamics of *S. haematobium* egg output over a period of 62 days after administration of a single dose of praziquantel in school-aged children. Additionally, we studied the post-treatment dynamics of infection-associated parameters, namely macro- and microhaematuria, proteinuria and leukocyturia.

## Methods

### Study area and population

The study was conducted between March and June 2010 in a primary school in the village of Grand Moutcho, located in the district of Agboville (geographical coordinates: 05°56'0'' N latitude and 04°13'0'' W longitude), approximately 75 km north of Abidjan, in southern Côte d’Ivoire. The climate in the area is tropical with two rainy seasons lasting from April to July and from mid-September to November. The study site was selected based on previous work conducted in Grand Moutcho that had shown a high endemicity of *S. haematobium*[30,31]*.* All children attending grades 3, 4, 5 and 6 in the primary school *Grand Moutcho I* were invited to participate. Prior to the current study, the village of Grand Moutcho had not been targeted by preventive chemotherapy with praziquantel against schistosomiasis.

### Ethical consideration and consent

The study protocol was approved by the institutional research commission of the Swiss Tropical and Public Health Institute (Basel, Switzerland) and received ethical clearance from the ethics commission in Basel (EKBB; reference no. 378/09), the ethics commission of the Albert-Ludwigs-University (Freiburg, Germany; reference no. 06/10), and the Comité National d’Ethique et de la Recherche (CNER) in Côte d’Ivoire (reference no. 1993 MSHP/CNER).

District health and education authorities, village leaders, teachers, children and parents/guardians were informed about the purpose, procedures and potential risk and benefits of the study and invited to approve their children’s participation in our study. A written informed consent was signed by a parent or guardian of eligible children at the beginning of the study. Children could withdraw from the study at any time without further obligation. All study medication was provided free of charge and administered by qualified medical personnel. At the end of the study, all children at the school were treated with a single oral dose of praziquantel (40 mg/kg using a dose pole) and albendazole (400 mg) [26,27].

### Sample size

The current study was planned to include approximately 100 school-aged children with a parasitologically confirmed *S. haematobium* infection for monitoring the post-treatment dynamics of *S. haematobium* egg output. This number was partially based on operational consideration (diagnostic work-up of urine samples at the individual collection days) to ascertain high quality results with the available resources. The sample size was considered large enough to study trends over time and to have only a small impact of single individuals on the overall outcomes.

### Study design and procedures

After approval of the study by district authorities, village leaders and the school director, the teachers were asked to prepare lists of the children attending their classes. To obtain our desired number of 100 *S. haematobium*-infected children, all children registered in grades 3–6 were invited to participate in the study. Parents/guardians were informed about the study procedures and questions arising were discussed in an open forum. Children who provided a written informed consent signed by their parent/guardian were eligible to participate in the baseline cross-sectional screening conducted in March 2010.

At the baseline cross-sectional survey, two urine samples were collected from each child over consecutive school days. Children were given 120 ml plastic containers during the morning break at 10:00 hours and asked to fill the container with their own urine. Samples were collected between 10:00 and 14:00 hours, labelled with unique identifiers and transferred to a field laboratory in Agboville town. Urine samples were worked up in the afternoon. First they were visually examined for the presence of blood (macrohaematuria), and then tested with reagent strips for microhaematuria, proteinuria and leukocyturia (Combur-7-Test**®**, Roche Diagnostics; Basel, Switzerland). Microhaematuria was detected above 5 erythrocytes/μl, proteinuria above 30 mg/dl and leukocyturia above 10 cells/μl. Next, urine samples were examined with the urine filtration method for the presence of *S. haematobium* eggs [14]. In brief, urine samples were vigorously shaken, and 10 ml of each sample were pressed through a 13-mm diameter small-meshed filter (20 μm) (Sefar AG; Heiden, Switzerland). Each filter was placed on a microscope slide labelled with the child’s identifier, and a drop of Lugol’s iodine solution was added before examination of the slide under a microscope by an experienced laboratory technician. The presence and number of *S. haematobium* eggs on each filter was recorded. For quality control, 10% of the slides were re-examined by a senior laboratory technician.

**Figure 1:**
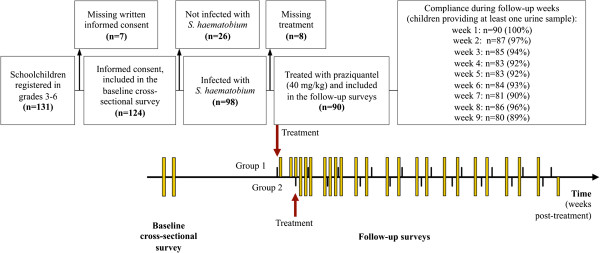
**Timeline of a study assessing the dynamics of *S. haematobium *egg output and associated infection parameters after praziquantel treatment, among school-aged children in south Côte d’Ivoire in 2010. **Flow chart showing the number of children invited to participate in the study, the children included in the baseline screening, and finally the overall compliance of children participating in the 62-day longitudinal surveillance to measure the dynamics of *S. haematobium *egg output and associated infection parameters after a single oral dose of praziquantel (40 mg/kg). The timeline shows the frequency of urine sampling (yellow bars) during the follow-up survey with the maximum number of urine samples collected per child during each follow-up week after treatment.

Children who had at least one *S. haematobium* egg on one of the two baseline survey days were invited for treatment and to participate in the follow-up surveys. Children infected with *S. haematobium* were treated with a single oral dose of praziquantel (40 mg/kg)*.* Those children who were not present on the first day of treatment were treated on a second treatment day, scheduled 5 days later. After drug administration children were followed-up over a period of 62 days. For this purpose, urine samples were always collected between 10:00 and 14:00 hours on 23 occasions. In the first two weeks we aimed to collect urine samples from each child on all school days (4 times a week). In the subsequent six weeks, we aimed to receive two samples per child per week. In the last week of the follow-up survey (week 9 post-treatment) only one sample was collected. The maximum number of times urine samples were collected per child during each follow-up week is shown in Figure 1.

As in the baseline survey, on each sampling day, urine samples were transferred to the laboratory and examined visually for the presence of macrohaematuria, tested with reagent strips and subjected to a urine filtration method for *S. haematobium* egg detection.

### Statistical analysis

Data were double entered in Microsoft Excel, checked for consistency and analysed in STATA version 10.0 (StataCorp; College Station, USA). The *S. haematobium* infection prevalence, defined as the percentage of children with *S. haematobium* eggs in urine, was calculated at baseline and at follow-up (on a daily or weekly basis). Whenever more than one urine sample was examined for a single child (i.e. at baseline and the weekly follow-up summary), a child was considered *S. haematobium-*positive if at least one egg was detected in at least one of the urine samples submitted in a specific week.

*S. haematobium* eggs counted on a filter are expressed as number of eggs/10 ml of urine. If a urine sample contained less than 10 ml, the egg count was adjusted to 10 ml. To assess the infection intensity before and on a weekly basis after treatment, the available egg count records per child were averaged. The *S. haematobium* infection intensity was classified into light (<50 eggs/10 ml of urine) and heavy (≥50 eggs/10 ml of urine), as defined by the World Health Organization (WHO) [26].

The group egg output intensity before and on a daily and weekly basis after treatment was determined by calculating the group’s geometric mean (GM; formula: GM (egg count +1) – 1) and arithmetic mean (AM) egg count values, including all available individual averaged egg counts.

Since praziquantel treatment was administered on two different days, daily follow-up results are presented for each of the two treatment groups separately. Weekly prevalence rates and AM and GM egg count values include combined data from both treatment groups.

For drug efficacy assessment, cure rate (CR; formula: [number of children excreting no *S. haematobium* eggs / number of children with confirmed infection before treatment] x 100) and egg reduction rate (ERR; formula AM: 1 – [AM egg count post-treatment / AM egg counts pre-treatment] x 100); formula GM: 1 – [GM egg count post-treatment / GM egg counts pre-treatment] x 100) were calculated for each time point post-treatment. Most former studies used GM as standard for determining ERR [29], but discussions are underway whether AM should be used instead. Hence, we present both approaches to render our results comparable to previously conducted and potential future studies.

Additionally, the weekly prevalence of infection-associated parameters (macrohaematuria, microhaematuria, proteinuria and leukocyturia) was calculated. When summarising the results at weekly intervals, a child was considered as overall positive for a parameter if a positive result was measured at any day of the weekly urine examination. Because samples were not tested with reagent strips on the last day of the survey, data for reagent strip testing and macroscopic examination is only available until week 8 post-treatment.

## Results

### Study cohort

A total of 131 schoolchildren were registered on the class lists of grades 3–6, provided by the teachers. As shown in Figure 1, 124 (94.7%) children had written informed consent, and hence, were eligible for the baseline cross-sectional survey. There were 62 girls (50.0%) and 62 boys. The mean age was 11 years with a range from 7 to 15 years.

*S. haematobium* infections were detected in 98 of the 124 children examined (79.0%), with equal prevalences among boys and girls. Heavy infection intensities were found in 34 of the infected children (34.7%), the remaining 64 infected children (65.3%) had light infections.

On the first treatment day, 27 children (group 1) were administered a single oral dose of praziquantel. Five days later, another 63 children (group 2) were treated. Eight *S. haematobium*-infected children were absent on both treatment days, and hence excluded from the follow-up surveys. These children received praziquantel at the end of the study. Demographic characteristics and baseline morbidity of our study cohort, stratified by the two treatment groups, are presented in Table 1.

**Table 1:** **Baseline characteristics of the 90 ** ***S. haematobium*-infected children included in the 62-day follow-up survey after treatment with a single dose of praziquantel (40 mg/kg)**

**Characteristics**	**Group 1**	**Group 2**	**Total**
No. of children	27	63	**90**
Sex			
Female n (%)		12 (44.4)	32 (50.8)	**44 (48.9)**
Male n (%)	15 (55.6)	31 (49.2)	**46 (51.1)**	
Age (years)				
Median	11	11	**11**	
Mean	11.3	10.9	**11.2**	
Range	7–15	8–15	**7–15**	
*S. haematobium *infection status at baseline				
Light infection n (<50 eggs/10 ml of urine)	21 (77.8)	36 (57.1)	**57 (63.3)**	
Heavy infection n (≥50 eggs/10 ml of urine)	6 (22.2)	27 (42.9)	**33 (36.7)**	
Mean *S. haematobium *egg counts (eggs/10 ml of urine)				
AM egg count (95% CI)	57 (16–98)	97 (66–128)	**85 (60–110)**	
GM egg count (95% CI)	11 (5–24)	41 (28–60)	**28 (20–40)**	

*AM*, arithmetic mean; *CI*, confidence interval; *GM*, geometric mean.

The study was carried out in the village of Grand Moutcho in south Côte d’Ivoire between March and June 2010. Children are stratified by treatment group.

### Dynamics of *S. haematobium* egg output after praziquantel administration

**Figure 2:**
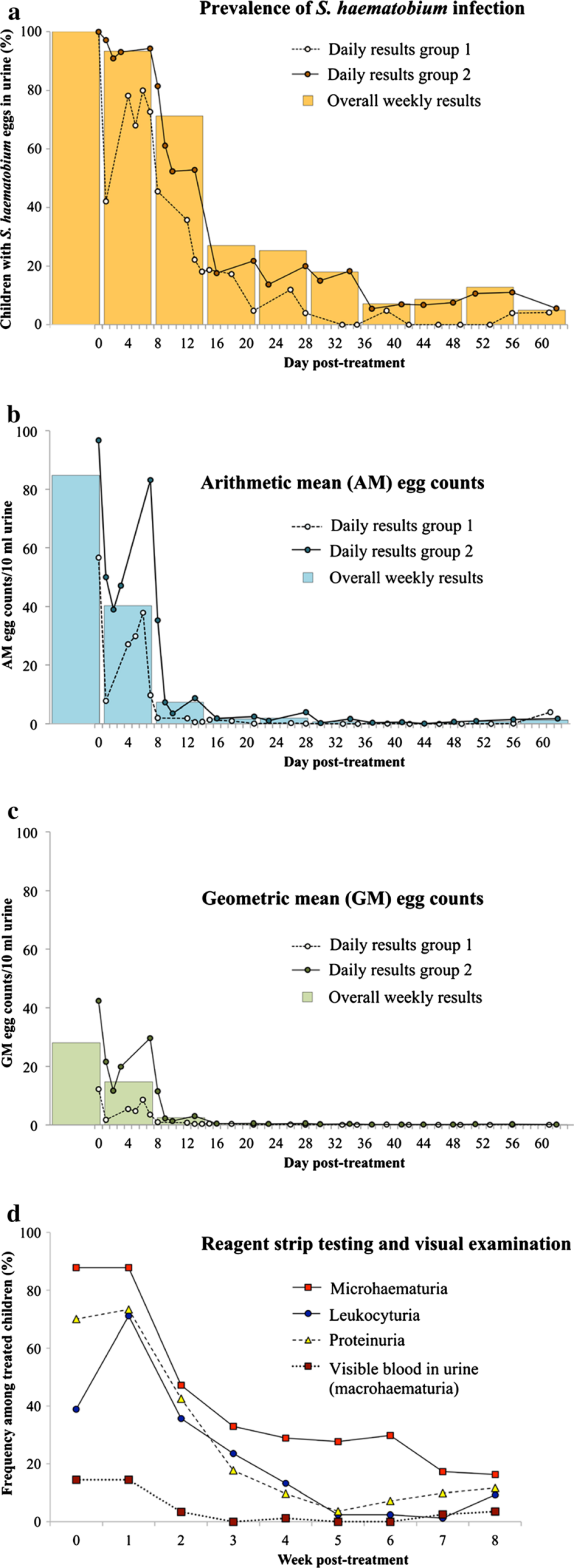
**Dynamics of *S. haematobium *egg output and associated infection parameters in two groups of children in southern Côte d’Ivoire after a single oral dose of praziquantel (40 mg/kg).** Results of urine filtration, reagent strip testing and visual examination of urine samples during the 62-day follow-up period, among 90 school-aged children with a parasitologically confirmed *S. haematobium *infection: (**a**) Frequency (%) of children with *S. haematobium *eggs in urine; (**b**) arithmetic mean (AM) egg counts per 10 ml of urine; (**c**) geometric mean (GM) egg counts per 10 ml of urine. Results are shown for each sampling day (dots) and summarised in weekly intervals (bars). (**d**) Weekly results of reagent strip testing and visual examination. Children were considered positive for a specific parameter during each follow-up week if they had at least one positive test result on one sampling day during the respective 7-day period.

In both groups the number of children with *S. haematobium* eggs in urine decreased after treatment with praziquantel. Figure 2a shows the prevalence of *S. haematobium* infection on each sampling day and is summarised in weekly intervals. The infection rate of 100% at baseline started to slowly decrease in week 1 post-treatment (93.3%), dropped considerably in weeks 2 (71.3%) and 3 (27.1%) and decreased further until week 6 (7.1%), followed by a slight increase in weeks 7 (8.6%), and 8 (12.8%). The lowest prevalence was observed in week 9 post-treatment (5.0%).

The decreasing slopes of daily and weekly group AM and GM egg count values from baseline to all follow-up days are presented as dots in Figures 2b and 2c, respectively. In both treatment groups, daily AM egg counts decreased considerably within the first two days post-treatment, from 56.8 and 96.8 eggs/10 ml of urine, respectively, to 7.8 and 39.0 eggs/10 ml of urine, respectively. However, until around day 7 post-treatment, AM egg counts increased again, to a maximum level of 37.9 and 83.2 eggs/10 ml of urine, respectively. Subsequently, AM egg counts dropped within a few days to low levels (1.9 and 3.6 eggs/10 ml of urine, respectively) around day 11 post-treatment. The pattern of GM egg count dynamics followed that of AM egg counts. From day 13 post-treatment onwards until the end of our observation period, the daily AM and GM egg counts remained very low, reaching ERRs between 95.9% and 100% on all sampling days in both groups.

The overall weekly AM egg count, shown as bars in Figures 2b and 2c, dropped from 84.8 eggs/10 ml of urine at baseline to 40.5 eggs/10 ml of urine in week 1 and 7.3 eggs/10 ml of urine in week 2 post-treatment, and continued to decrease to 1.7, 1.9 and 0.7 eggs/10 ml of urine in weeks 3, 4 and 5 post-treatment, respectively. In weeks 6 and 7, a minimum of 0.3 eggs/10 ml of urine was observed. A slight increase of the weekly counts to 0.8 and 1.3 eggs/10 ml of urine was observed in the last two weeks of the survey. The weekly overall GM decreased parallel to the AM until week 6 and remained constant at 0.1 eggs/10 ml of urine until the end of our observation period.

### Dynamics of infection-related parameters post-treatment

As shown in Figure 2d, the overall weekly prevalence of the four infection-related parameters (i.e. macrohaematuria, microhaematuria, proteinuria and leukocyturia) decreased over the course of the treatment intervention. At the baseline cross-sectional survey, 13 of the 90 *S. haematobium*-infected children had visible blood in their urine, hence, a prevalence of 14.4%. The number did not change over the first week post-treatment, but decreased to 3 (3.5%) in week 2 post-treatment. Between weeks 3 and 6 post-treatment, no child presented with macrohaematuria, with the exception of one child with visible blood in urine at week 4. After week 6 post-treatment, blood was observed in the urine of four children. Among them was one girl (13 years) with light infection intensity and one boy (13 years) with heavy infection intensity that remained until the end of the survey. One girl (15 years) and one boy (11 years) had visible blood in urine without passing eggs.

The prevalence of microhaematuria was also stable from baseline to week 1 post-treatment (87.8%), but then dropped sharply to 47.1% in week 2 post-treatment and 32.9% in week 3. Microhaematuria remained at around 30% until week 6 post-treatment. Towards the end of the study, microhaematuria further decreased to a minimum of 16.3%, eight weeks after treatment.

The prevalence of proteinuria and leukocyturia among all treated children increased from 70.0% and 38.9% at baseline to 73.3% and 71.1%, respectively, in week 1 post-treatment. Subsequently, both parameters decreased gradually until week 5 post-treatment (3.6% and 2.4%, respectively). The prevalence of proteinuria then increased to 11.6% in week 8 post-treatment, while the prevalence of leukocyturia reached a minimum of 1.2% in week 7 post-treatment, then increased to 9.3% in week 9 post-treatment.

## Discussion

Praziquantel is the main pillar of schistosomiasis control. The number of countries administering praziquantel within the frame of preventive chemotherapy programmes to school-aged children and other at-risk populations is increasing [28,32]. Since no real alternative drug against schistosomiasis is currently available [15,21,24], there is a need to closely monitor the efficacy of praziquantel, particularly in areas where high coverage is reached and treatment is repeated frequently. Standard protocols for drug efficacy assessment and monitoring do not yet exist, but are needed, so that the potential development of drug resistance can be detected early on [26,29]. For recommendation of the most appropriate time point to assess CR and ERR of praziquantel against *S. haematobium* infection, we studied the dynamics of *S. haematobium* egg output and associated infection parameters over a 62-day period following treatment with a standard dose of 40 mg/kg praziquantel in a group of school-aged children in south Côte d’Ivoire.

### *S. haematobium* infection prevalence and egg output post-treatment

We found that both CR and ERR were highly dependent on the time of measurement post-treatment. The CR was highest in week 6 (between day 36 and day 42) post-treatment (92.9%). The AM and GM ERR increased sharply in the first two weeks after treatment and showed a constantly high level from week 3 post-treatment onwards (>97.5%). These findings are in line with observations from Gabon, where a significant reduction in daily *S. haematobium* egg counts between day 3 and day 9 post-treatment was observed, among schoolchildren treated with praziquantel and observed for 35 days post-treatment [33]. A study from South Africa found a high GM ERR (95.3%), but only a moderate CR (57.9%) three weeks post-treatment [34]. In Cameroon, observed CRs at weeks 3 and 6 were 40-50% and 83%, respectively, after praziquantel administration [35], corroborating our findings. Hence, we recommend that CRs be assessed at around day 36 post-treatment, whereas ERRs can be assessed considerably earlier, from day 15 post-treatment onwards. However, it should be noted that our results were obtained in a high prevalence setting without prior praziquantel treatment, and hence care is indicated in generalizing our findings to other areas with different infection intensity profiles that have been subjected to prior large-scale administration of praziquantel.

The tendency of finding increased egg output and a higher number of *S. haematobium*-positive children in weeks 7 and 8 post-treatment is possibly due to schistosomula that were not affected by praziquantel, and hence have further developed, matured and paired up in the weeks after treatment to start oviposition [29]. Reinfection might have also occurred, but it is unlikely that one finds *S. haematobium* eggs in the urine of newly infected individuals, as it takes more than 60 days from infection to oviposition [36]. Since our study started during the dry season in March 2010, when the transmission of *S. haematobium* should be low, the effect of juvenile worms might not be as strong as during high transmission season [37]. An increase in *S. mansoni* egg excretion already four weeks post-treatment was observed in a preceding study conducted in western Côte d’Ivoire that followed a similar design as the work presented here [38]. Since the development of *S. haematobium* worms takes considerably longer than that of *S. mansoni*, whilst the period of insusceptibility is the same in both species, egg output might be seen at a later stage for *S. haematobium* than for *S. mansoni*[22,23,39]. However, it has been suggested that insusceptibility of schistosomula to praziquantel is less pronounced in *S. haematobium* than in *S. mansoni*[23]. A study from Cameroon did not find lower CRs 9 weeks post-treatment compared to 6 weeks post-treatment and therefore suggested good efficacy of praziquantel against all stages of *S. haematobium*[35]. In view of our findings and those of others, we suggest that CR should be assessed before week 7 post-treatment, and we encourage further research on drug susceptibility of immature *S. haematobium* worms.

Notably, the slight decrease in prevalence observed in week 9 of our study is likely due to the fact that only one instead of two urine samples per child were collected. Indeed, previous research carried out in Côte d’Ivoire, South Africa and Zimbabwe has shown that the more intense the sampling effort post-treatment, the lower the observed CR [34,40-42]. Future standard operating procedures should therefore include advice on the sampling strategy for drug efficacy monitoring. We suggest the microscopic examination of at least two urine samples, collected between 10:00 and 14:00 over consecutive days, for determining praziquantel efficacy in terms of CR.

It is important to note that *S. haematobium* and *S. mansoni* often co-exist [43-45]. In such co-endemic settings, mainly for operational and practical reasons, it would be useful to have a single time point for assessment of praziquantel treatment efficacy. According to our data pertaining to *S. haematobium* and recommendations made by Scherrer and colleagues for *S. mansoni*[38], the optimal time points for assessment of CRs differ between the two schistosome species (i.e. week 3 post-treatment for *S. mansoni* and week 6 post-treatment for *S. haematobium*). As a compromise, perhaps, CRs of both infections could be measured in weeks 4 or 5 post-treatment. However, it needs to be considered that at this time point, *S. haematobium* infection prevalence might not be at a minimum, and hence care is indicated with regard to drug efficacy evaluation and monitoring of potential resistance development. If morbidity control rather than elimination of schistosomiasis is the goal of the treatment intervention, assessing ERRs in both species at one time point will be a useful strategy. Indeed, egg counts of both species have been shown to be constantly low in both species from week 3 post-treatment onwards [38]. We conclude that ERRs can be assessed at a single time point from day 14 post-treatment onwards.

### *S. haematobium* egg output during the first days post-treatment

The observation that *S. haematobium* egg counts first dropped rapidly within one day but increased again until the end of the first week post-treatment before they dropped sharply and remained at low levels from week 3 onwards seems an artefact at first sight. However, this phenomenon has also been described for *S. mansoni* egg output after treatment with praziquantel [38]. One possible explanation is that not all adult worms die immediately after treatment but some might be paralysed and reproduce heavily before dying within weeks 1–3 post-treatment. Another explanation might be that praziquantel has a direct effect on the host tissue, leading to a decreased release of eggs into the bladder. Praziquantel might also have a direct effect on *S. haematobium* eggs. Indeed, it has been described that praziquantel in very high doses can lead to hatching of eggs in the tissue [46]. If this finding is translatable to regular treatment doses with praziquantel, the temporary “disappearance” of schistosome eggs might not be due to dying adult worms but rather due to early hatching of miracidia. Further research is needed to elucidate the exact mechanisms of praziquantel on eggs, different developmental stages of the worms and reactions in the human body.

### Other infection parameters post-treatment

In addition to studying egg output dynamics using the urine filtration method, we assessed the evolution of *S. haematobium*-associated infection parameters after treatment with praziquantel. As shown in previous studies, the prevalence of microhaematuria, proteinuria and leukocyturia decreased after treatment with praziquantel [47-50]. Proteinuria and leukocyturia showed a transient minimum around week 5 post-treatment. Both parameters increased again towards the end of our 2-month observation period, which might point to new eggs being excreted by surviving worms and triggering inflammation and lesions in the urinary tract. Microhaematuria remained more common than the other parameters until the end of the study and many children were still passing blood in urine without excreting *S. haematobium* eggs. Towards the end of our study, four children had macrohaematuria, but they did not pass any or only very few eggs in their urine. Possible explanations for the remaining macrohaematuria are as follows. First, two of the children were girls, aged 13 and 15 years, and hence the observed blood might be due to menstruation. Second, one 13-year-old boy had likely high exposure levels, since he presented with a heavy infection before treatment and had a very quick increase in egg counts shortly after treatment.

Our findings of lasting egg-negative haematuria after treatment are in line with a study from Kenya where the prevalence of microhaematuria decreased more slowly than the prevalence of eggs detected by microscopy and where it took 4 to 6 months until the lowest levels were reached [51,52]. Lesions in the urinary tract with small bleeds might still be present even after egg output has terminated. Indeed, ultrasound examination has shown that urinary tract pathology resolves at a slower rate than reduction in egg output during the first months after treatment [52,53]. As for microhaematuria, a decrease of proteinuria and leukocyturia 5 to 6 weeks post-treatment might serve as an additional parameter for drug efficacy and morbidity reduction assessment in settings of high endemicity where initial prevalences of microhaematuria, proteinuria and leukocyturia are elevated [18,54].

## Conclusion

Praziquantel was highly efficacious against *S. haematobium* among school-aged children in an area with high endemicity in south Côte d’Ivoire where no prior treatment campaigns took place. Prevalence and intensity of infection, haematuria, proteinuria and leukocyturia all decreased sharply. Praziquantel efficacy against urogenital schistosomiasis in terms of ERR should be measured at the earliest from week 3 post-treatment. The optimal time point for CR assessment in our study was at week 6 post-treatment. In settings where *S. haematobium* and *S. mansoni* co-exist, drug efficacy evaluation at a single time point, perhaps at weeks 4 to 5 post-treatment, should be envisaged. For an accurate assessment of the CR, multiple urine samples collected over consecutive days are needed. Reagent strip testing, indicating a decrease of microhaematuria, proteinuria and leukocyturia after treatment might serve as a valuable tool for the evaluation of treatment success. We encourage other researchers to study further the exact mechanisms of praziquantel on all stages of *S. haematobium* in the human body, in order to explain the phenomenon of a transitory drop of egg counts during the first days after treatment.

## Competing interests

The authors declare that they have no competing interests.

## Authors’ contributions

KS, EKN and JU designed the study; KS, SJK, JTC, IM and LKL implemented the study; KS, SJK and JTC managed the data; KS, JH and JU analysed and interpreted the data; KS, SK and JU wrote the paper. WVK, EKN and JU supervised the different phases of the study. All authors read, revised and approved the final manuscript.
